# Prevalence of Disorders Recorded in Dogs Attending Primary-Care Veterinary Practices in England

**DOI:** 10.1371/journal.pone.0090501

**Published:** 2014-03-04

**Authors:** Dan G. O′Neill, David B. Church, Paul D. McGreevy, Peter C. Thomson, Dave C. Brodbelt

**Affiliations:** 1 Veterinary Epidemiology, Economics and Public Health, Royal Veterinary College, London, United Kingdom; 2 Small Animal Medicine and Surgery Group, Royal Veterinary College, London, United Kingdom; 3 Faculty of Veterinary Science, University of Sydney, Sydney, New South Wales, Australia; University of Missouri, United States of America

## Abstract

Purebred dog health is thought to be compromised by an increasing occurence of inherited diseases but inadequate prevalence data on common disorders have hampered efforts to prioritise health reforms. Analysis of primary veterinary practice clinical data has been proposed for reliable estimation of disorder prevalence in dogs. Electronic patient record (EPR) data were collected on 148,741 dogs attending 93 clinics across central and south-eastern England. Analysis in detail of a random sample of EPRs relating to 3,884 dogs from 89 clinics identified the most frequently recorded disorders as otitis externa (prevalence 10.2%, 95% CI: 9.1–11.3), periodontal disease (9.3%, 95% CI: 8.3–10.3) and anal sac impaction (7.1%, 95% CI: 6.1–8.1). Using syndromic classification, the most prevalent body location affected was the head-and-neck (32.8%, 95% CI: 30.7–34.9), the most prevalent organ system affected was the integument (36.3%, 95% CI: 33.9–38.6) and the most prevalent pathophysiologic process diagnosed was inflammation (32.1%, 95% CI: 29.8–34.3). Among the twenty most-frequently recorded disorders, purebred dogs had a significantly higher prevalence compared with crossbreds for three: otitis externa (P = 0.001), obesity (P = 0.006) and skin mass lesion (P = 0.033), and popular breeds differed significantly from each other in their prevalence for five: periodontal disease (P = 0.002), overgrown nails (P = 0.004), degenerative joint disease (P = 0.005), obesity (P = 0.001) and lipoma (P = 0.003). These results fill a crucial data gap in disorder prevalence information and assist with disorder prioritisation. The results suggest that, for maximal impact, breeding reforms should target commonly-diagnosed complex disorders that are amenable to genetic improvement and should place special focus on at-risk breeds. Future studies evaluating disorder severity and duration will augment the usefulness of the disorder prevalence information reported herein.

## Introduction

The domestic dog (*Canis lupus familiaris*) has become integral to modern human family life, with the UK dog population estimated to be 8–10 million [1], [2], [3] and 24–31% of UK households estimated to own at least one dog [1], [2]. Although humans benefit from dog ownership both physically [4], [5] and mentally [6], [7], it is increasingly questioned whether modern breeding practices have allowed dog health and welfare to derive comparable benefits [8], [9]. Although the dog is now the most phenotypically diverse mammal at a species level [10], genetic diversity has been greatly reduced within modern breeds [11] because of breeding practices that include closed stud books [12], structured inbreeding [11] and reproductive dominance of popular sires [13]. Additionally, selection pressure within breeds towards phenotypic exaggeration driven by breed standards [8], have increased the potential for conformation-associated disease [14]. Each of the 50 most popular breeds in the UK has at least one reported conformational predisposition to disease [15] and almost 400 non-conformational inherited disorders have been identified [16]. Conversely, implicit acceptance of the statement that purebred dogs are plagued with many inherited diseases [17] has contributed to a widespread belief that crossbred dogs are substantially healthier than purebreds [18].

Following claims in the BBC documentary *Pedigree Dogs Exposed* that purebred dog health was deteriorating because of inbreeding and ill-advised breed standards [19], three major reports concurred that pedigree breeding practices did impose welfare costs on dogs but, more crucially, concluded that a critical data gap on disorder prevalence information in UK dogs constrained effective reforms [20], [21], [22]. Prevalence data have been published on only 1% of inherited disorders affecting popular UK dog breeds [23]. Effective welfare reform of pedigree dog-breeding must be underpinned by scientifically valid prioritisation of disorders based on reliable and comparable prevalence data [12], [24]. However, differing case definitions, study populations, geographical locations, data quality and data collection periods between published studies, combined with substantial data gaps, have constrained efforts to prioritise disorders in domestic dogs [9]. Application of health data collected via a single national surveillance system has been proposed for effective disorder prioritisation, with the critical first step being the generation of reliable disorder prevalence values [12].

Systematised collection, mergence and analysis of electronic patient record (EPR) data from primary-care veterinary practices has been proposed for generation of reliable prevalence data relating to the overall dog population [12], [20]. Contemporaneous recording of clinical information by veterinary health professionals during episodes of care for every patient treated minimises selection and recall biases in primary-care practice EPR data [20]. By contrast, referral caseloads may show selection bias towards more complicated disorders [25], questionnaire surveys may incur selection, recall and misclassification biases [26], and pet insurance data are limited by selection bias emerging from age restrictions, financial excesses and owner attributes [27].

This study aimed to use a database of merged primary-care practice EPRs to estimate the prevalence of the most frequently recorded disorders and syndromes in dogs attending primary-care veterinary practices in England. The study further aimed to evaluate associations between the occurrence of common disorders with purebred/crossbred status and with popular breeds. It was hypothesised that purebred dogs have a higher prevalence of common disorders compared with crossbred dogs.

## Materials and Methods

Ethics statement: Ethics approval was granted by the RVC Ethics and Welfare Committee (reference number 2010 1076).

The VetCompass Animal Surveillance project collates de-identified EPR data from primary-care veterinary practices in the UK for epidemiological research [28]. The current study included data collected from all clinics within the Medivet Veterinary Group, a large network of integrated veterinary practices covering central and south-eastern England [29]. Practitioners recorded summary diagnosis terms from an embedded standard nomenclature, the VeNom codes [30], at episodes of clinical care. EPR data were extracted from practice management systems (PMSs) using integrated clinical queries [31] and uploaded to a secure structured query language (SQL) database. Information collected included patient demographic (animal identification number, species, breed, date of birth, sex, neuter status, insurance status, microchip number and weight) and clinical information (free-form text clinical notes, VeNom summary diagnosis terms and treatment, with relevant dates) data fields.

The study sampling frame included all dogs that had at least one EPR (clinical note, weight recording or treatment dispensed) recorded within the VetCompass Animal Surveillance database from September 1, 2009 to March 31, 2013. Sample size calculations estimated that, from a study population of 140,000 dogs, a sample of 3,648 animals was required to represent a disorder with 2.5% expected frequency with a precision of 0.5% at a 95% confidence level [32].

A random sample of dogs was selected from the overall sampling frame using an online random number generator (www.random.org). Clinical notes and VeNom summary diagnosis terms recorded during the study period were reviewed in detail, and the most definitive diagnostic term recorded for each disorder diagnosed within individual dogs was manually coded using the most appropriate VeNom term. Elective (e.g. neutering) or prophylactic (e.g. vaccination) clinical events were not included. Multiple counting of disorder events for ongoing cases was avoided by including recurring diagnoses of ongoing conditions only once (e.g. repeated events of otitis externa) and by including only the final diagnosis term recorded in cases with diagnosis revision over time (e.g. following clinical work-up or trial therapy), based on the assumption that diagnostic accuracy increased over time [33]. The parent term was used for disorders that encompassed multiple child terms [34] (e.g. a parent term *road traffic accident* (RTA) may have multiple child terms such as *laceration*, *fracture* and *hypovolaemic shock*). Disorder events that were aetiologically independent despite sharing the same disorder term name (e.g. novel traumatic events) were included separately. No distinction was made between pre-existing and incident disorder presentations. Disorders described within the clinical notes using presenting sign terms (e.g. ‘vomiting and diarrhoea’), but without a formal clinical diagnostic term being recorded, were included using the first sign listed (e.g. vomiting). Dental disorders were included only if surgical or medical intervention were recommended.

Recognisable single breeds [35] were grouped as ‘purebred’ while all other dogs were grouped as ‘crossbred’. Purebreds were further categorised by Kennel Club (KC) breed-recognition (recognised/not recognised) and KC breed group (gundog, hound, pastoral, terrier, toy, utility, working) [36]. Neuter status was defined by the final EPR neuter value and was combined with sex to create four categories: female entire, female neutered, male entire and male neutered. Insurance and microchip values characterized the existence of a positive status at any time during the study period. The maximum bodyweight (kg) recorded for dogs aged over one year was categorised into seven groups (<10.0, 10.0–19.9, 20.0–29.9, 30.0–39.9, 40.0–49.9, ≥50.0, and ‘no recorded weight’). The age (years) at the final EPR was categorised into five groups (<1.0, 1.0–2.9, 3.0–5.9, 6.0–9.9, ≥10.0). Time contributed to the study for each dog was calculated as the period from the date of the earliest EPR to the date of the latest EPR. The date and manner (euthanasia or non-assisted) [37] of deaths recorded during the study were identified.

VeNom diagnostic terms for all recorded disorders were extracted and mapped to three systems of terms for analysis: diagnosis-level precision, mid-level precision and syndromic classification. Diagnosis-level terms were one-to-one descriptors of the original extracted terms at the maximal diagnostic precision recorded within the clinical notes (e.g. *inflammatory bowel disease* would remain as *inflammatory bowel disease*). Mid-level precision terms were one-to-one descriptors of original diagnosis terms defined at a general level of diagnostic precision (e.g. *inflammatory bowel disease* would map to *enteropathy*). Syndromic classification used three taxonomic groupings: body location, organ system and pathophysiologic process. The number of syndromic terms that could be mapped from each original diagnostic term was not limited.

Study data were exported from the VetCompass database to a spreadsheet (Microsoft Office Excel 2007, Microsoft Corp.) for checking and cleaning before further export to Stata Version 11.2 (Stata Corporation) for statistical analyses. Demographic variables were described statistically for the overall study population and the sample group. Prevalence values with 95% confidence intervals (CI) were tabulated for the twenty most prevalent diagnosis-level and mid-level disorders and for all syndromic terms, and were reported across all sampled dogs, purebreds only and crossbreds only. Prevalence values for purebred and crossbred dogs were compared statistically using the chi-squared test with Holm-adjusted P-values to account for multiple testing effects [38]. Statistical significance was set at the 5% level. The CI estimates were derived from standard errors based on approximation to the normal distribution for disorders with ten or more events recorded [39], but the Wilson approximation method was used for disorders with fewer than ten events recorded [40]. Prevalence (95% CI) values for the twenty most prevalent diagnosis-level and mid-level disorders and for all syndromic terms were similarly derived, reported and compared for popular breeds and crossbreds (popular breeds had ≥100 dogs in the sample group).

## Results

The overall population comprised 148,741 dogs attending 93 clinics across central and south-eastern England. Demographic examination of dogs with information available indicated that 117,179 (78.9%) were purebred, 71,002 (48.0%) were female, 61,120 (41.1%) were neutered, 43,435 (29.2%) were insured and 41,071 (27.6%) were microchipped. The median weight was 18.2 kg (interquartile range (IQR): 9.4–29.0, range: 0.68–105.0) and the median age was 4.5 years (IQR: 1.6–8.7, range: 0.0–27.4) (Table 1).

**Table 1:** Demographic information for sampled (n = 3,884) and overall study population (n = 148,741) dogs attending primary veterinary practices in England.

Variable	Category	Sample: No. (%)	Population: No. (%)
**Sex/neuter**	Female entire	981 (25.4)	40,514 (27.4)
	Female neutered	836 (21.6)	30,488 (20.6)
	Male entire	1,152 (29.8)	46,459 (31.4)
	Male neutered	899 (23.2)	30,635 (20.7)
**Microchip**	Not microchipped	2,733 (70.4)	107,670 (72.4)
	Microchipped	1,151 (29.6)	41,071 (27.6)
**Purebred status**	Crossbred	797 (20.6)	31,354 (21.1)
	Purebred	3,079 (79.4)	117,179 (78.9)
**Popular breeds**	Crossbreed	797 (20.5)	31,354 (21.1)
	Labrador Retriever	339 (8.7)	13,328 (9.0)
	Staffordshire Bull Terrier	334 (8.6)	12,212 (8.2)
	Jack Russell Terrier	262 (6.8)	10,006 (6.7)
	Cocker Spaniel	133 (3.4)	5,579 (3.8)
	German Shepherd Dog	132 (3.4)	5,314 (3.6)
	Yorkshire Terrier	127 (3.3)	4,880 (3.3)
	Border Collie	104 (2.7)	3,997 (2.7)
	Other named breeds	1,656 (42.6)	62,071 (41.7)
**KC** a **- breed** b	Not KC-recognised	306 (9.9)	11,717 (10.0)
	KC-recognised	2,773 (90.1)	105,462 (90.0)
**KC** a **group** c	Gundog	737 (26.6)	28,832 (27.3)
	Hound	178 (6.4)	6,505 (6.2)
	Pastoral	284 (10.2)	11,530 (10.9)
	Terrier	561 (20.2)	21,481 (20.4)
	Toy	474 (17.1)	17,215 (16.3)
	Utility	330 (11.9)	11,573 (11.0)
	Working	209 (7.5)	8,326 (7.9)
**Weight (kg)**	No recorded weight	1,260 (32.4)	52,308 (35.2)
	<10.0	769 (19.8)	26,786 (18.0)
	10.0–19.9	695 (17.9)	25,278 (17.0)
	20.0–20.99	579 (14.9)	21,869 (14.7)
	30.0–30.9	390 (10.0)	15,255 (10.3)
	40.0–40.9	130 (3.4)	5,118 (3.4)
	≥50.0	61 (1.6)	2,127 (1.4)
**Age (years)**	<1.0	588 (15.2)	24,915 (16.8)
	1.0–2.9	791 (20.4)	30,747 (20.7)
	3.0–5.9	877 (22.6)	33,500 (22.5)
	6.0–9.9	811 (20.9)	30,811 (20.7)
	≥10.0	814 (21.0)	28,664 (19.3)
**Insurance**	Non-insured	2,658 (68.4)	105,306 (70.8)
	Insured	1,226 (31.6)	43,435 (29.2)

a KC The Kennel Club.

b Percentage values based on purebred only.

c Percentage values based on KC-recognised dogs only.

The study sample comprised 3,884 dogs attending 89 clinics. Of dogs with information available, 3,079 (79.4%) were purebred, 1,817 (47.0%) were female, 1,735 (44.7%) were neutered, 1,226 (31.6%) were insured and 1,151 (29.6%) were microchipped. The median weight was 17.3 kg (IQR: 9.1–28.4, range: 1.3–100.6) and the median age was 4.8 years (IQR: 1.8–9.1, range: 0.0–21.24). The most popular seven breeds accounted for 1,431 (36.8%) of the study sample dogs (Table 1). Of the sampled dogs, 378 (9.7%) died during the study period, with a median (IQR, range) age at death of 12.3 years (9.2–14.4, 0.0–21.0) and 336 (88.9%) deaths involving euthanasia. Overall, 2,945 (75.8%) dogs had at least one disorder diagnosed, with the remainder having no disorders diagnosed during the study period. The median (IQR, range) number of disorders diagnosed per dog was 1.0 (1.0–3.0, 0.0–21.0). The median (IQR, range) time contributed to the study per dog was 0.7 years (0.0–3.5, 0.0–1.9). The sample and study populations were similar across all measures assessed.

Among the sampled dogs, 8,025 unique disorder events were recorded encompassing 430 distinct diagnosis-level disorder terms. The most prevalent diagnosis-level disorders recorded were otitis externa (number of events: 396, prevalence: 10.2%, 95% CI: 9.1–11.3), periodontal disease (361, 9.3%, 95% CI: 8.3–10.3), anal sac impaction (277, 7.1%, 95% CI: 6.1–8.1) and overgrown nails (276, 7.1%, 95% CI: 6.1–8.2). Purebred dogs had a significantly higher prevalence compared with crossbreds for three of the twenty most-prevalent diagnosis-level disorders: otitis externa (P = 0.001), obesity (P = 0.006) and skin mass lesion (P = 0.033) (Table 2). The prevalence of five of the twenty most-prevalent diagnosis-level disorders differed statistically significantly between popular breeds: periodontal disease (P = 0.002), overgrown nails (P = 0.004), degenerative joint disease (P = 0.005), obesity (P = 0.001) and lipoma (P = 0.003) (Table 3).

**Table 2:** Prevalence results for the most frequent disorders recorded in dogs, purebreds only and crossbreds only that attended primary veterinary practices in England.

	Overall			Purebred		Crossbred		
Disorder	No.	Prev^a^%	95% CI^b^	Prev^a^%	95% CI^b^	Prev^a^%	95% CI^b^	P-value
**Otitis externa**	396	10.2	9.1–11.3	11.2	10.0–12.4	6.5	4.7–8.3	**0.001**
**Periodontal disease**	361	9.3	8.3–10.3	9.4	8.2–10.5	9.2	7.4–11.0	1.000
**Anal sac impaction**	277	7.1	6.1–8.1	7.1	6.0–8.1	7.5	5.7–9.4	1.000
**Overgrown nails**	276	7.1	6.1–8.2	6.9	5.8–8.0	8.0	6.1–9.9	1.000
**Degenerative joint disease**	256	6.6	5.7–7.5	6.4	5.3–7.4	7.5	5.7–9.4	1.000
**Diarrhoea**	249	6.4	5.5–7.4	6.8	5.6–8.0	4.9	3.4–6.4	0.255
**Obesity**	238	6.1	5.2–7.1	6.7	5.6–7.9	3.9	2.3–5.5	**0.006**
**Traumatic injury**	214	5.5	4.7–6.4	5.5	4.4–6.5	5.7	3.6–7.7	1.000
**Conjunctivitis**	192	4.9	4.1–5.8	5.2	4.2–6.2	4.1	2.8–5.5	1.000
**Vomiting**	159	4.1	3.3–4.9	4.0	3.1–4.9	4.5	3.0–6.0	1.000
**Heart murmur**	153	3.9	3.3–4.5	4.1	3.5–4.7	3.4	2.1–4.7	1.000
**Lipoma**	137	3.5	2.8–4.2	3.5	2.7–4.2	3.8	2.7–4.9	1.000
**Dermatitis**	134	3.5	2.8–4.1	3.5	2.8–4.3	3.1	1.9–4.4	1.000
**Skin hypersensitivity**	113	2.9	2.3–3.5	3.2	2.5–3.9	1.8	0.9–2.6	0.116
**Skin mass**	110	2.8	2.3–3.4	3.2	2.6–3.8	1.5	0.6–2.4	**0.033**
**Claw injury**	103	2.7	2.1–3.2	2.6	2.0–3.2	2.6	1.5–3.8	1.000
**Behavioural**	99	2.6	2.1–3.0	2.6	2.1–3.1	2.4	1.4–3.4	1.000
**Gastroenteritis**	99	2.6	2.0–3.1	2.4	1.9–2.9	3.1	2.0–4.3	1.000
**Dog bite injury**	97	2.5	1.9–3.1	2.4	1.7–3.1	2.9	1.8–4.0	1.000
**Laceration**	92	2.4	1.8–2.9	2.5	1.8–3.1	2.0	1.1–2.9	0.446

P-values (Holm-adjusted) represent comparison between purebreds and crossbreds.

a Prev prevalence.

b 95% CI 95% confidence interval.

**Table 3:** Prevalence results for frequent disorders recorded in popular breeds (number of dogs) from 3,884 randomly sampled dogs attending primary veterinary practices in England.

	Prevalence percentage (95% confidence interval)								
Disorder	Crossbred (797)	Labrador Retriever (339)	Staffordshire Bull Terrier (334)	Jack Russell Terrier (262)	Cocker Spaniel (133)	German Shepherd Dog (132)	Yorkshire Terrier (127)	Border Collie (104)	P-Value
**Otitis externa**	6.5 (4.7–8.3)	11.8 (8.8–15.7)	9.9 (7.1–13.6)	6.9 (4.4–10.6)	8.3 (4.7–14.2)	11.4 (7.0–17.9)	7.9 (4.3–13.9)	1.9 (0.5–6.7)	0.084
**Periodontal disease**	9.2 (7.4–11.0)	3.2 (1.8–5.7)	2.4 (1.2–4.7)	9.5 (6.6–13.7)	12.8 (8.1–19.5)	4.5 (2.1–9.6)	25.2 (18.6–33.4)	6.7 (3.3–13.3)	**0.002**
**Anal sac impaction**	7.5 (5.7–9.4)	4.7 (2.9–7.5)	3.3 (1.9–5.8)	6.9 (4.4–10.6)	12.0 (7.5–18.6)	6.1 (3.1–11.5)	6.3 (3.2–11.9)	2.9 (1.0–8.1)	0.066
**Overgrown nails**	8.0 (6.1–9.9)	6.5 (4.3–9.6)	3.9 (2.3–6.5)	13.7 (10.1–18.4)	2.3 (0.8–6.4)	1.5 (0.4–5.4)	15.0 (9.8–22.2)	1.0 (0.2–5.3)	**0.004**
**Degenerative joint disease**	7.5 (5.9–9.6)	11.5 (8.5–15.3)	5.4 (3.4–8.4)	4.2 (2.4–7.4)	1.5 (0.4–5.3)	6.8 (3.6–12.5)	1.6 (0.4–5.6)	11.5 (6.7–19.1)	**0.005**
**Diarrhoea**	4.9 (3.4–6.4)	8.3 (5.8–11.7)	4.8 (3.0–7.6)	4.6 (2.6–7.8)	9.8 (5.8–16.0)	8.3 (4.7–14.3)	5.5 (2.7–10.9)	7.7 (4.0–14.5)	1.000
**Obesity**	3.9 (2.3–5.5)	13.0 (9.8–17.0)	6.0 (3.9–9.1)	5.3 (3.2–8.8)	8.3 (4.7–14.2)	2.3 (0.8–6.5)	0.8 (0.1–4.3)	6.7 (3.3–13.3)	**0.001**
**Traumatic injury**	5.7 (3.6–7.7)	5.3 (3.4–8.2)	4.5 (2.7–7.3)	6.1 (3.8–9.7)	5.3 (2.6–10.5)	4.6 (2.1–9.6)	3.2 (1.2–7.8)	4.8 (2.1–10.8)	1.000
**Conjunctivitis**	4.1 (2.8–5.5)	4.1 (2.5–6.8)	5.1 (3.2–8.0)	4.2 (2.4–7.4)	6.8 (3.6–12.4)	0.0 (0.0–2.8)	7.1 (3.8–12.9)	4.8 (2.1–10.8)	1.000
**Vomiting**	4.5 (3.0–6.0)	3.8 (2.3–6.5)	3.9 (2.3–6.5)	5.7 (3.5–9.2)	2.3 (0.8–6.4)	4.6 (2.1–9.6)	3.2 (1.2–7.8)	1.9 (0.5–6.7)	1.000
**Heart murmur**	3.4 (2.1–4.7)	1.5 (0.6–3.4)	2.7 (1.4–5.0)	3.8 (2.1–6.9)	3.8 (1.6–8.5)	1.5 (0.4–5.4)	7.1 (3.8–12.9)	4.8 (2.1–10.8)	0.837
**Lipoma**	3.8 (2.7–4.9)	9.1 (6.5–12.7)	2.1 (1.0–4.3)	2.7 (1.3–5.4)	6.0 (3.1–11.4)	1.5 (0.4–5.4)	2.1 (0.0–2.9)	5.8 (2.7–12.0)	**0.003**
**Dermatitis**	3.1 (1.9–4.4)	1.5 (0.6–3.4)	3.6 (2.1–6.2)	3.4 (1.8–6.4)	3.0 (1.2–7.5)	3.0 (1.2–7.5)	4.7 (2.2–9.9)	6.7 (3.3–13.3)	1.000
**Skin hypersensitivity**	1.8 (0.9–2.6)	3.8 (2.3–6.5)	5.1 (3.2–8.0)	3.1 (1.6–5.9)	1.5 (0.4–5.3)	3.0 (1.2–7.5)	3.2 (1.2–7.8)	2.9 (1.0–8.1)	1.000
**Skin mass**	1.5 (0.6–2.4)	3.2 (1.8–5.7)	3.9 (2.3–6.5)	2.3 (1.1–4.9)	3.8 (1.6–8.5)	3.0 (1.2–7.5)	2.4 (0.8–6.7)	3.0 (1.0– 8.1)	1.000
**Claw injury**	2.6 (1.5–3.8)	3.8 (2.3–6.5)	3.6 (2.1–6.2)	2.7 (1.3–5.4)	2.3 (0.8–6.4)	3.0 (1.2–7.5)	3.9 (1.7–8.9)	2.9 (1.0–8.1)	1.000
**Undesirable behaviour**	2.4 (1.4–3.4)	3.0 (1.6–5.3)	2.7 (1.4–5.0)	1.5 (0.6–3.9)	3.0 (1.2–7.5)	7.6 (4.2–13.4)	2.4 (0.8–6.7)	5.8 (2.7–12.0)	0.208
**Gastro**–**enteritis**	3.1 (2.0–4.3)	4.4 (2.7–7.3)	1.5 (0.6–3.5)	1.9 (0.8–4.4)	3.0 (1.2–7.5)	0.8 (0.1–4.2)	3.9 (1.7–8.9)	3.9 (1.5–9.5)	1.000
**Dog bite injury**	2.9 (1.8–4.0)	1.5 (0.6–3.4)	3.0 (1.6–5.4)	3.8 (2.1–6.9)	3.8 (1.6–8.5)	1.5 (0.4–5.4)	0.0 (0.0–2.9)	1.0 (0.2–5.3)	1.000
**Laceration**	2.0 (1.2–3.2)	3.5 (2.0–6.1)	2.4 (1.2–4.7)	2.7 (1.3–5.4)	3.0 (1.2–7.5)	0.8 (0.1–4.2)	1.6 (0.4–5.6)	2.9 (1.0–8.1)	1.000

P-values (Holm-adjusted) represent comparison between breeds.

Within 54 mid-level diagnosis terms, the most prevalent disorders were enteropathic (*n* = 692, prevalence: 17.8%, 95% CI: 16.0–19.6), dermatological (602, 15.5%, 95% CI: 13.9–17.1), musculoskeletal (457, 11.8%, 95% CI: 10.6–12.9) and aural (426, 11.0%, 95% CI: 9.8–12.2). Purebred dogs showed a significantly higher prevalence than crossbreds for four of the twenty most-prevalent mid-level disorders: dermatological (P = 0.004), aural (P = 0.001), ophthalmological (P = 0.032) and obesity (P = 0.009) (Table 4). Statistically significant differences in prevalence values were shown between the most popular breeds in eight of the twenty most-frequent mid-level disorders: musculoskeletal (P = 0.002), claw/nail (P = 0.008), dental (P = 0.007), neoplastic (P = 0.001), anal sac (P = 0.006), obesity (P = 0.004), cardiac (P = 0.005) and brain (P = 0.003) (Table 5).

**Table 4:** Prevalence results for the most frequent mid-level disorders recorded in dogs, purebreds only and crossbreds only that attended primary veterinary practices in England.

	Overall			Purebred		Crossbred		
Mid-level disorder	No.	Prev^a^%	95% CI^b^	Prev^a^%	95% CI^b^	Prev^a^%	95% CI^b^	P-value
**Enteropathic**	692	17.8	16.0–19.6	17.7	15.8–19.7	18.3	15.4–21.2	1.000
**Dermatological**	602	15.5	13.9–17.1	16.5	14.6–18.4	11.9	10.0–13.9	**0.004**
**Musculoskeletal**	457	11.8	10.6–12.9	11.2	9.8–12.6	14.1	11.8–16.3	0.130
**Aural**	426	11.0	9.8–12.2	12.0	10.7–13.3	7.2	5.3–9.0	**0.001**
**Ophthalmological**	406	10.5	9.1–11.8	11.1	9.7–12.6	7.9	6.1–9.7	**0.032**
**Claw/nail**	400	10.3	9.1–11.5	10.1	8.8–11.5	10.9	9.0–12.9	1.000
**Dental**	386	9.9	8.8–11.1	10.0	8.8–11.2	9.8	7.9–11.7	1.000
**Neoplastic**	367	9.5	8.2–10.7	9.6	8.2–10.9	9.2	7.2–11.1	1.000
**Traumatic injury (not incl. bites)**	351	9.0	8.0–10.1	9.1	7.8–10.3	8.9	6.6–11.2	1.000
**Anal sac**	337	8.7	7.5–9.8	8.6	7.3–9.9	9.0	7.1–11.0	1.000
**Obesity**	238	6.1	5.2–7.1	6.7	5.6–7.9	3.9	2.3–5.5	**0.009**
**Mass lesion**	235	6.1	5.2–6.9	6.4	5.3–7.4	4.9	3.4–6.4	0.726
**Behavioural**	233	6.0	5.3–6.85	5.8	4.9–6.7	6.9	5.1–8.7	1.000
**Upper respiratory tract**	223	5.7	4.9–6.5	5.6	4.6–6.6	6.4	4.6–8.2	1.000
**Cardiac**	219	5.6	4.8–6.5	5.9	5.0–6.7	4.9	3.1–6.7	1.000
**Parasitic**	172	4.4	3.8–5.1	4.2	3.5–5.0	5.3	3.7–6.8	1.000
**Congenital**	171	4.4	3.7–5.1	4.6	3.7–5.4	3.9	2.6–5.2	1.000
**Bite injury**	148	3.8	3.0–4.6	3.7	2.9–4.6	4.1	2.8–5.5	1.000
**Urinary**	126	3.2	2.7–3.8	3.4	2.7–4.1	2.8	1.6–3.9	1.000
**Brain**	122	3.1	2.5–3.7	3.2	2.6–3.8	3.1	1.9–4.4	1.000

P-values (Holm-adjusted) represent comparison between purebreds and crossbreds.

a Prev prevalence.

b 95% CI 95% confidence interval.

**Table 5:** Prevalence results for frequent mid-level disorders recorded in popular breeds (number of dogs) attending primary veterinary practices in England.

	Prevalence percentage (95% confidence interval)								
Mid-level disorder	Crossbred (797)	Labrador Retriever (339)	Staffordshire Bull Terrier (334)	Jack Russell Terrier (262)	Cocker Spaniel (133)	German Shepherd Dog (132)	Yorkshire Terrier (127)	Border Collie (104)	P-value
**Enteropathic**	18.3 (15.4–21.2)	22.7 (18.6–27.5)	13.2 (10.0–17.2)	15.3 (11.4–20.1)	18.8 (13.1–26.3)	20.5 (14.5–28.1)	16.5 (11.1–24.0)	17.3 (11.2–25.7)	1.000
**Dermatological**	11.9 (10.0–13.9)	16.8 (13.2–21.2)	14.7 (11.38–18.9)	13.0 (9.4–17.6)	9.8 (5.8–16.0)	18.9 (13.2–26.5)	18.1 (12.4–25.7)	18.3 (12.0–26.8)	0.715
**Musculoskeletal**	14.1 (11.8–16.3)	16.2 (12.7–20.5)	8.4 (5.9–11.9)	7.3 (4.7–11.1)	3.0 (1.2–7.5)	16.7 (11.3–24.0)	6.3 (3.2–11.9)	16.4 (10.5–24.6)	**0.002**
**Aural**	7.2 (5.3–9.0)	12.1 (9.0–16.0)	11.1 (8.1–14.9)	7.6 (5.0–11.5)	9.0 (5.2–15.1)	11.4 (7.0–17.9)	7.9 (4.3–13.9)	4.8 (2.1–10.8)	0.828
**Ophthalmological**	7.9 (6.1–9.7)	6.8 (4.6–10.0)	8.1 (5.6–11.5)	8.0 (5.3–11.9)	12.0 (7.5–18.7)	2.3 (0.8–6.5)	12.6 (7.9–19.5)	12.5 (7.5–20.2)	0.261
**Claw/nail**	10.9 (9.0–12.9)	10.9 (8.0–14.7)	7.5 (5.1–10.8)	14.9 (11.1–19.7)	5.3 (2.6–10.5)	5.3 (2.6–10.5)	19.7 (13.7–27.5)	5.8 (2.7–12.0)	**0.008**
**Dental**	9.8 (7.9–11.7)	3.8 (2.3–6.5)	3.0 (1.6–5.4)	11.5 (8.1–15.9)	12.8 (8.1–19.5)	5.3 (2.6–10.5)	25.2 (18.5–33.4)	7.7 (4.0–14.5)	**0.007**
**Neoplastic**	9.2 (7.2–11.1)	14.8 (11.4–18.9)	6.6 (4.4–9.8)	4.6 (2.6–7.8)	13.5 (8.7–20.4)	4.6 (2.1–9.6)	6.3 (3.2–11.9)	8.7 (4.6–15.6)	**0.001**
**Traumatic injury (not bites or claw)**	8.9 (6.6–11.2)	11.2 (8.3–15.0)	7.88 (5.4–11.2)	9.2 (6.2–13.3)	10.5 (6.4–16.9)	6.1 (3.1–11.5)	3.9 (1.7–8.9)	9.6 (5.3–16.8)	1.000
**Anal sac**	9.0 (7.1–11.0)	5.9 (3.9–8.9)	3.6 (2.1–6.2)	9.9 (6.9–14.1)	13.5 (8.7–20.4)	6.8 (3.6–12.5)	6.3 (3.2–11.9)	2.9 (1.0–8.1)	**0.006**
**Obesity**	3.9 (2.3–5.5)	12.98 (9.81–16.98)	6.0 (3.9–9.1)	5.3 (3.2–8.8)	8.3 (4.7–14.2)	2.3 (0.8–6.5)	0.8 (0.1–4.3)	6.7 (3.3–13.3)	**0.004**
**Mass lesion**	4.9 (3.4–6.4)	8.26 (5.78–11.68)	6.6 (4.4–9.8)	5.0 (2.9–8.3)	6.8 (3.6–12.4)	6.1 (3.1–11.5)	7.9 (4.3–13.9)	8.7 (4.6–15.6)	1.000
**Behavioural**	6.9 (5.1–8.7)	4.7 (2.9–7.5)	5.1 (3.2–8.0)	7.6 (5.0–11.5)	6.8 (3.6–12.4)	12.9 (8.2–19.7)	3.9 (1.7–8.9)	8.7 (4.6–15.6)	0.460
**Upper respiratory tract**	6.4 (4.6–8.2)	6.2 (4.1–9.3)	6.3 (4.2–9.4)	5.7 (3.5–9.2)	2.3 (0.8–6.4)	3.0 (1.2–7.5)	7.1 (3.8–12.9)	2.9 (1.0–8.1)	1.000
**Cardiac disorder**	4.9 (3.1–6.7)	1.5 (0.6–3.4)	3.0 (1.6–5.4)	6.5 (4.1–10.1)	4.5 (2.1–9.5)	1.5 (0.4–5.4)	10.2 (6.1–16.7)	5.8 (2.7–12.0)	**0.005**
**Parasitic**	5.3 (3.7–6.8)	3.5 (2.0–6.1)	4.8 (3.0–7.6)	3.4 (1.8–6.4)	8.3 (4.7–14.2)	2.3 (0.8–6.5)	4.7 (2.2–9.9)	1.9 (0.5–6.7)	1.000
**Congenital**	3.9 (2.6–5.2)	2.4 (1.2–4.6)	2.1 (1.0–4.3)	3.8 (2.2–6.9)	3.8 (1.6–8.5)	0.8 (0.1–4.2)	6.3 (3.2–11.9)	1.9 (0.5–6.7)	1.000
**Bite injury**	4.1 (2.8–5.5)	3.8 (2.3–6.5)	4.29 (2.5–6.9)	5.0 (2.9–8.3)	4.5 (2.1–9.5)	2.3 (0.8–6.5)	1.6 (0.4–5.6)	1.9 (0.5–6.7)	1.000
**Urinary**	2.8 (1.6–3.9)	4.7 (2.9–7.5)	2.4 (1.2–4.6)	1.9 (0.8–4.4)	3.0 (1.2–7.5)	3.0 (1.2–7.5)	2.4 (0.8–6.7)	3.9 (1.5–9.5)	1.000
**Brain**	3.1 (1.9–4.4)	3.2 (1.8–5.7)	0.6 (0.2–2.2)	2.3 (1.1–4.9)	3.0 (1.2–7.5)	4.6 (2.1–9.6)	1.6 (0.4–5.6)	9.6 (5.3–16.8)	**0.003**

P–values (Holm–adjusted) represent comparison between breeds. (*n* = 3,884).

Syndromic classification analysis indicated that the most prevalent body locations affected in dogs were the head-and-neck (*n* = 1,273, prevalence = 32.8%, 95% CI: 30.7–34.9), abdomen (993, 25.6%, 95% CI: 23.6–27.5) and limb (679, 17.5%, 95% C: 15.9–19.1). Purebreds had significantly higher prevalence values compared with crossbreds for two of the eight body locations: head-and-neck (P = 0.003) and tail (P = 0.038) disorders. The most prevalent organ systems affected were the integument (1,408, 36.3%, 95% CI: 33.9–38.6), digestive (1,144, 29.5%, 95% CI: 27.5–31.5) and musculoskeletal (573, 14.8%, 95% CI: 13.8–16.0) (Table 6). Purebreds had significantly higher prevalence values than crossbreds for two of fifteen organ systems, namely integument (P = 0.001) and auditory (P = 0.002) (Table 6). The most prevalent pathophysiologic processes recorded were inflammation (1,246, 32.1%, 95% CI: 29.8–34.3), mass/swelling (625, 16.1%, 95% CI: 14.6–17.6) and traumatic (557, 14.3%, 95% CI: 12.8–15.9). Purebreds had significantly higher prevalence values than crossbreds for two of twenty-one pathophysiological processes: inflammatory (P = 0.006) and nutritional (P = 0.0014) disorders (Table 7). Statistically significant differences in prevalence values between the most popular breeds were shown for 5/8 body location terms, 5/15 organ system terms and 5/21 pathophysiologic processes (Tables 8, 9 &10).

**Table 6:** Prevalence of syndromic disorders affecting body location and organ system recorded in overall dogs, purebreds only and crossbreds only that attended primary veterinary practices in England.

	Overall				Purebred		Crossbred		
	No.		Prev^a^%	95% CI^b^	Prev^a^%	95% CI^b^	Prev^a^%	95% CI^b^	P–value
**Body Location**									
Head/neck	1,273		32.8	30.7–34.9	34.0	31.7–36.2	28.5	24.9–32.0	**0.003**
Abdomen	993		25.6	23.6–27.5	25.9	23.7–28.0	24.6	21.5–27.7	1.000
Limb	679		17.5	15.9–19.1	17.3	15.5–19.1	18.3	15.7–20.9	1.000
Anus/perineum	359		9.2	8.1–10.4	9.1	7.8–10.5	9.8	7.6–12.0	1.000
Thorax	353		9.1	8.1–10.1	9.2	8.1–10.4	8.7	6.5–10.8	1.000
Vertebral column	78		2.0	1.5–2.5	2.0	1.5–2.6	2.0	1.0–3.0	1.000
Pelvis	33		0.9	0.6–1.2	0.9	0.7–1.4	0.5	0.2–1.3	0.684
Tail	21		0.5	0.4–0.8	0.7	0.5–1.0	0.0	0.0–0.5	**0.038**
**Organ system**									
Integument	1,408	36.3		33.9–38.6	37.6	35.0–40.2	31.4	28.0–34.7	**0.001**
Digestive	1,144	29.5		27.5–31.5	29.4	27.2–31.6	30.0	26.6–33.3	1.000
Musculoskeletal	573	14.8		13.5–16.0	14.1	12.6–15.6	17.3	14.8–19.8	0.110
Connective/Soft tissue	503	13.0		11.6–14.3	13.2	11.6–14.7	12.3	10.2–14.4	1.000
Ocular	447	11.5		10.2–12.8	12.2	10.6–13.7	9.2	7.2–11.1	0.057
Auditory	437	11.3		10.0–12.5	12.3	11.0–13.6	7.4	5.5–9.3	**0.002**
Nervous	301	7.8		6.8–8.7	7.7	6.7–8.7	7.9	6.2–9.6	1.000
Respiratory	273	7.0		6.2–7.9	7.0	6.0–8.1	7.2	5.2–9.1	1.000
Cardiovascular	241	6.2		5.3–7.1	6.5	5.5–7.4	5.3	3.5–7.1	1.000
Urinary	227	5.8		5.1–6.6	5.9	4.9–6.8	5.8	4.1–7.5	1.000
Reproductive	184	4.7		4.1–5.4	4.7	4.0–5.5	4.9	3.5–6.3	1.000
Endocrine	72	1.9		1.5–2.3	1.8	1.3–2.3	2.1	1.2–3.1	1.000
Haematopoietic	53	1.4		1.0–1.7	1.4	1.0–1.8	1.3	0.5–2.1	1.000
Hepatobiliary	29	0.8		0.5–1.1	0.9	0.6–1.3	0.1	0.0–0.7	0.088
Lymphatic	26	0.7		0.5–1.0	0.6	0.4–1.0	0.9	0.4–1.8	1.000

P–values (Holm–adjusted) represent comparison between purebreds and crossbreds.

a Prev prevalence.

b 95% CI 95% confidence interval.

**Table 7:** Prevalence of syndromic disorders related to pathophysiologic processes recorded in overall dogs, purebreds only and crossbreds only that attended primary veterinary practices in England.

	Overall			Purebred		Crossbred			
Pathophysiologic process	No.	Prev^a^%	95% CI^b^	Prev^a^%	95% CI^b^	Prev^a^%	95% CI^b^	P–value	
**Inflammation**	1,246	32.1	29.8–34.3	33.2	30.7–35.7	28.1	25.1–31.2		**0.006**
**Mass/swelling**	625	16.1	14.6–17.6	16.7	15.0–18.4	14.1	11.8–16.3		0.222
**Traumatic**	557	14.3	12.8–15.9	14.3	12.7–16.0	14.3	11.6–17.0		1.000
**Degenerative**	411	10.6	9.4–11.8	10.4	9.0–11.7	11.4	9.1–13.8		1.000
**Infectious**	388	10.0	9.0–11.0	10.3	9.1–11.4	9.0	6.9–11.2		1.000
**Neoplastic**	336	8.7	7.6–9.8	8.6	7.3–9.8	9.0	7.2–10.9		1.000
**Congenital/developmental**	332	8.6	7.4–9.7	8.9	7.6–10.2	7.3	5.6–9.2		0.870
**Nutritional**	320	8.2	7.1–9.4	8.9	7.5–10.2	5.9	4.3–7.5		**0.014**
**Behavioural**	262	6.8	5.9–7.6	6.5	5.5–7.4	7.9	6.0–9.8		1.000
**Hereditary**	232	6.0	5.1–6.9	6.2	5.1–7.3	5.3	3.5–7.0		1.000
**Parasitic**	221	5.7	5.0–6.4	5.5	4.6–6.3	6.7	5.0–8.4		1.000
**Iatrogenic**	150	3.9	3.3–4.5	3.7	3.1–4.4	4.4	2.9–5.9		1.000
**Foreign body**	109	2.8	2.3–3.3	2.8	2.3–3.4	2.8	1.6–3.9		1.000
**Death**	65	1.7	1.2–2.2	1.6	1.1–2.1	2.1	1.2–3.1		1.000
**Intoxicative**	49	1.3	1.0–1.7	1.3	1.0–1.8	1.1	0.6–2.1		1.000
**Haemostatic**	38	1.0	0.7–1.3	1.1	0.8–1.5	0.5	0.2–1.3		0.496
**Immune–mediated**	38	1.0	0.7–1.3	1.1	0.8–1.5	0.5	0.2–1.3		0.620
**Allergic**	35	0.9	0.7–1.3	0.9	0.6–1.3	0.9	0.4–1.8		1.000
**Thermoregulatory**	17	0.4	0.3–0.7	0.4	0.2–0.7	0.6	0.3–1.5		1.000
**Metabolic**	8	0.2	0.1–0.4	0.2	0.1–0.4	0.3	0.1–0.9		1.000
**Effusion**	1	0.0	0.0–0.2	0.0	0.0–0.2	0.0	0.0–0.5		1.000

P–values (Holm–adjusted) represent comparison between purebreds and crossbreds.

a Prev prevalence.

b 95% CI 95% confidence interval.

**Table 8:** Prevalence of syndromic diagnoses affecting body location recorded in crossbred dogs and popular breeds (number of dogs) from 3,884 randomly sampled dogs attending primary veterinary practices in England.

	Prevalence percentage (95% confidence interval)								
**Body Location**	Crossbred (797)	Labrador Retriever (339)	Staffordshire Bull Terrier (334)	Jack Russell Terrier (262)	Cocker Spaniel (133)	German Shepherd Dog (132)	Yorkshire Terrier (127)	Border Collie (104)	**P–Value**
**Head/neck**	28.5 (24.9–32.0)	28.6 (24.1–33.6)	24.0 (19.7–28.8)	30.5 (25.3–36.4)	33.1 (25.7–41.5)	22.7 (16.4–30.6)	43.3 (35.0–52.0)	35.6 (27.0–45.1)	**0.006**
**Abdomen**	24.6 (21.5–27.7)	32.4 (27.7–37.6)	21.0 (16.9–25.6)	21.0 (16.5–26.3)	27.1 (20.2–35.2)	25.8 (19.1–33.8)	20.5 (14.4–28.3)	30.8 (22.7–40.2)	**0.045**
**Limb**	18.3 (15.7–20.9)	20.4 (16.4–25.0)	14.1 (10.7–18.2)	20.2 (15.8–25.5)	7.5 (4.1–13.3)	13.6 (8.8–20.5)	22.0 (15.7–30.0)	16.3 (10.5–24.6)	**0.036**
**Anus/perineum**	9.8 (7.6–12.0)	6.2 (4.1–9.3)	3.9 (2.3–6.5)	9.9 (6.9–14.1)	15.0 (10.0–22.1)	9.1 (5.3–15.2)	7.1 (3.8–12.9)	3.8 (1.5–9.5)	**0.001**
**Thorax**	8.7 (6.5–10.8)	6.5 (4.3–9.6)	6.0 (3.9–9.1)	8.8 (5.9–12.8)	6.0 (3.1–11.4)	3.0 (1.2–7.5)	13.4 (8.5–20.4)	6.7 (3.3–13.2)	0.294
**Vertebral column**	2.0 (1.0–3.0)	1.5 (0.6–3.4)	0.3 (0.1–1.7)	1.1 (0.4–3.3)	3.8 (1.6–8.5)	1.5 (0.4–5.4)	0.8 (0.1–4.3)	2.9 (1.0–8.1)	1.000
**Pelvis**	0.5 (0.2–1.3)	0.6 (0.2–2.1)	0.6 (0.2–2.2)	0.0 (0.0–1.4)	0.0 (0.0–2.8)	0.0 (0.0–2.8)	1.6 (0.4–5.6)	1.0 (0.2–5.2)	1.000
**Tail**	0.0 (0.0–0.5)	2.4 (1.2–4.6)	0.3 (0.1–1.7)	0.0 (0.0–1.4)	1.5 (0.4–5.3)	0.0 (0.0–2.8)	0.0 (0.0–2.9)	1.0 (0.2–5.2)	**0.002**

P–values (Holm–adjusted) represent comparison between breeds.

**Table 9:** Prevalence of syndromic diagnoses affecting organ system recorded in crossbred dogs and popular breeds (number of dogs) from 3,884 randomly sampled dogs attending primary veterinary practices in England.

	Prevalence percentage (95% confidence interval)									
**Organ system**	Crossbred (797)	Labrador Retriever (339)	Staffordshire Bull Terrier (334)	Jack Russell Terrier (262)	Cocker Spaniel (133)	German Shepherd Dog (132)	Yorkshire Terrier (127)		Border Collie (104)	**P–Value**
**Integument**	31.4 (28.0–34.7)	39.2 (34.2–44.5)	36.2 (31.3–41.5)	34.0 (28.5–39.9)	33.8 (26.3–42.2)	34.8 (27.3–43.3)	42.5 (34.3–51.2)	29.8 (21.9–39.2)		0.816
**Digestive**	30.0 (26.6–33.3)	29.8 (25.2–34.9)	19.2 (15.3–23.7)	28.6 (23.5–34.4)	36.1 (28.4–44.5)	27.3 (20.4–35.4)	44.1 (35.8–52.8)	26.9 (19.3–36.2)		**0.002**
**Musculoskeletal**	17.3 (14.8–19.8)	19.2 (15.3–23.7)	9.6 (6.9–13.2)	9.5 (6.5–13.7)	6.8 (3.6–12.4)	18.9 (13.2–26.5)	8.7 (4.9–14.8)	22.1 (15.2–31.0)		**0.005**
**Connective/Soft tissue**	12.3 (10.2–14.4)	16.2 (12.7–20.5)	9.9 (7.1–13.6)	9.5 (6.5–13.7)	14.3 (9.3–21.2)	5.3 (2.6–10.5)	9.4 (5.5–15.8)	17.3 (11.2–25.7)		0.060
**Ocular**	9.2 (7.2–11.1)	9.1 (6.5–12.7)	8.7 (6.1–12.2)	8.8 (5.9–12.8)	12.8 (8.1–19.5)	2.3 (0.8–6.5)	13.4 (8.5–20.4)	14.4 (8.9–22.4)		0.203
**Auditory**	7.4 (5.5–9.3)	12.4 (9.3–16.3)	11.1 (8.1–14.9)	8.4 (5.6–12.4)	10.5 (6.4–16.9)	11.4 (7.0–17.9)	7.9 (4.3–13.9)	5.8 (2.7–12.0)		1.000
**Nervous**	7.9 (6.2–9.6)	8.3 (5.8–11.7)	3.0 (1.6–5.4)	5.7 (3.5–9.2)	9.0 (5.2–15.1)	12.9 (8.2–19.7)	3.1 (1.2–7.8)	15.4 (9.7–23.5)		**0.003**
**Respiratory**	7.2 (5.2–9.1)	8.0 (5.5–11.3)	6.9 (4.6–10.1)	7.3 (4.7–11.0)	3.0 (1.2–7.5)	3.8 (1.6–8.6)	8.7 (4.9–14.8)	3.8 (1.5–9.5)		1.000
**Cardiovascular**	5.3 (3.5–7.1)	1.5 (0.6–3.4)	3.3 (1.8–5.8)	7.6 (5.0–11.5)	5.3 (2.6–10.5)	1.5 (0.4–5.4)	11.0 (6.7–17.7)	6.7 (3.3–13.2)		**0.001**
**Urinary**	5.8 (4.1–7.5)	5.3 (3.4–8.2)	3.6 (2.1–6.2)	4.6 (2.6–7.8)	6.8 (3.6–12.4)	4.5 (2.1–9.6)	6.3 (3.2–11.9)	6.7 (3.3–13.2)		1.000
**Reproductive**	4.9 (3.5–6.3)	2.7 (1.4–5.0)	6.0 (3.9–9.1)	5.0 (2.9–8.3)	5.3 (2.6–10.5)	2.3 (0.8–6.5)	3.9 (1.7–8.9)	1.0 (0.2–5.2)		1.000
**Endocrine**	2.1 (1.2–3.1)	1.5 (0.6–3.4)	1.2 (0.5–3.0)	2.3 (1.1–4.9)	0.0 (0.0–2.8)	0.8 (0.1–4.2)	2.4 (0.8–6.7)	1.9 (0.5–6.7)		1.000
**Haematopoietic**	1.3 (0.7–2.3)	2.1 (1.0–4.2)	1.2 (0.5–3.0)	0.4 (0.1–2.1)	1.5 (0.4–5.3)	1.5 (0.4–5.4)	0.0 (0.0–2.9)	0.0 (0.0–3.6)		1.000
**Hepatobiliary**	0.1 (0.0–0.7)	1.8 (0.8–3.8)	0.0 (0.0–1.1)	0.4 (0.1–2.1)	0.0 (0.0–2.8)	0.0 (0.0–2.8)	0.0 (0.0–2.9)	3.8 (1.5–9.5)		**0.004**
**Lymphatic**	0.9 (0.4–1.8)	0.6 (0.2–2.1)	0.6 (0.2–2.2)	0.4 (0.1–2.1)	0.8 (0.1–4.1)	0.0 (0.0–2.8)	0.0 (0.0–2.9)	1.0 (0.2–5.2)		1.000

P–values (Holm–adjusted) represent comparison between breeds.

**Table 10:** Prevalence of syndromic diagnoses relating to pathophysiologic processes recorded in crossbred and popular breeds (number of dogs) attending primary veterinary practices in England.

	Prevalence percentage (95% confidence interval)								
**Pathophysiologic process**	Crossbred (797)	Labrador Retriever (339)	Staffordshire Bull Terrier (334)	Jack Russell Terrier (262)	Cocker Spaniel (133)	German Shepherd Dog (132)	Yorkshire Terrier (127)	Border Collie (104)	**P–Value**
**Inflammation**	28.1 (25.1–31.2)	37.8 (32.8–43.0)	29.6 (25.0–34.7)	25.2 (20.3–30.8)	27.8 (20.9–36.0)	32.6 (25.2–41.0)	35.4 (27.7–44.1)	27.9 (20.2–37.2)	0.120
**Mass/swelling**	14.1 (11.8–16.3)	23.3 (19.1–28.1)	14.1 (10.7–18.2)	11.1 (7.8–15.4)	20.3 (14.3–27.9)	12.1 (7.6–18.8)	14.2 (9.2–21.3)	24.0 (16.8–33.1)	**0.004**
**Traumatic**	14.3 (11.6–17.0)	18.3 (14.5–22.8)	14.1 (10.7–18.2)	14.5 (10.8–19.3)	16.5 (11.2–23.8)	11.4 (7.0–17.9)	7.1 (3.8–12.9)	15.4 (9.7–23.5)	1.000
**Degenerative**	11.4 (9.1–13.8)	15.6 (12.2–19.9)	7.5 (5.1–10.8)	7.6 (5.0–11.5)	4.5 (2.1–9.5)	9.8 (5.8–16.1)	6.3 (3.2–11.9)	18.3 (12.0–26.8)	**0.001**
**Infectious**	9.0 (6.9–11.2)	13.9 (10.6–17.9)	7.8 (5.4–11.2)	8.0 (5.3–11.9)	9.0 (5.2–15.1)	10.6 (6.4–17.0)	7.9 (4.3–13.9)	13.5 (8.2–21.3)	0.990
**Neoplastic**	9.0 (7.2–10.9)	15.3 (11.9–19.6)	6.3 (4.1–9.4)	4.6 (2.6–7.8)	12.8 (8.1–19.5)	2.3 (0.8–6.5)	3.9 (1.7–8.9)	9.6 (5.3–16.8)	**0.003**
**Congenital**	7.3 (5.6–9.2)	5.0 (3.2–7.9)	4.8 (3.0–7.6)	6.5 (4.1–10.1)	7.5 (4.1–13.3)	6.1 (3.1–11.5)	11.0 (6.7–17.7)	5.8 (2.7–12)	1.000
**Nutritional**	5.9 (4.3–7.5)	16.5 (12.9–20.8)	7.5 (5.1–10.8)	6.9 (4.4–10.6)	9.8 (5.8–16.0)	3.8 (1.6–8.6)	2.4 (0.8–6.7)	9.6 (5.3–16.8)	**0.002**
**Behavioural**	7.9 (6.0–9.8)	5.0 (3.2–7.9)	6.6 (4.4–9.8)	8.8 (5.9–12.8)	6.8 (3.6–12.4)	13.6 (8.8–20.5)	3.9 (1.7–8.9)	8.7 (4.6–15.6)	0.400
**Hereditary**	5.3 (3.5–7.0)	4.4 (2.7–7.2)	3.3 (1.8–5.8)	3.4 (1.8–6.4)	3.0 (1.2–7.5)	7.6 (4.2–13.4)	11.8 (7.3–18.6)	2.9 (1.0–8.1)	**0.025**
**Parasitic**	6.7 (5.0–8.4)	6.2 (4.1–9.3)	5.7 (3.7–8.7)	5.0 (2.9–8.3)	9.8 (5.8–16.0)	3.0 (1.2–7.5)	5.5 (2.7–10.9)	2.9 (1.0–8.1)	1.000
**Iatrogenic**	4.4 (2.9–5.9)	4.4 (2.7–7.2)	3.0 (1.6–5.4)	4.2 (2.4–7.4)	3.8 (1.6–8.5)	4.5 (2.1–9.6)	4.7 (2.2–9.9)	4.8 (2.1–10.8)	1.000
**Foreign body**	2.8 (1.6–3.9)	3.2 (1.8–5.7)	1.2 (0.5–3.0)	2.7 (1.3–5.4)	2.3 (0.8–6.4)	2.3 (0.8–6.5)	0.0 (0.0–2.9)	3.8 (1.5–9.5)	1.000
**Death**	2.1 (1.2–3.1)	1.8 (0.8–3.8)	1.5 (0.6–3.5)	0.8 (0.2–2.7)	0.8 (0.1–4.1)	2.3 (0.8–6.5)	3.1 (1.2–7.8)	4.8 (2.1–10.8)	1.000
**Intoxicative**	1.1 (0.6–2.1)	1.5 (0.6–3.4)	0.9 (0.3–2.6)	1.5 (0.6–3.9)	0.8 (0.1–4.1)	0.0 (0.0–2.8)	1.6 (0.4–5.6)	1.0 (0.2–5.2)	1.000
**Haemostatic**	0.5 (0.2–1.3)	1.5 (0.6–3.4)	1.8 (0.8–3.9)	1.1 (0.4–3.3)	0.0 (0.0–2.8)	0.8 (0.1–4.2)	0.0 (0.0–2.9)	2.9 (1.0–8.1)	1.000
**Immune–mediated**	0.5 (0.2–1.3)	0.0 (0.0–1.1)	0.3 (0.1–1.7)	0.8 (0.2–2.7)	2.3 (0.8–6.4)	0.0 (0.0–2.8)	2.4 (0.8–6.7)	1.0 (0.2–5.2)	0.189
**Allergic**	0.9 (0.4–1.8)	2.1 (1.0–4.2)	0.6 (0.2–2.2)	0.8 (0.2–2.7)	0.8 (0.1–4.1)	0.8 (0.1–4.2)	1.6 (0.4–5.6)	0.0 (0.0–3.6)	1.000
**Thermoregulatory**	0.6 (0.3–1.5)	0.0 (0.0–1.1)	0.6 (0.2–2.2)	0.8 (0.2–2.7)	0.8 (0.1–4.1)	0.0 (0.0–2.8)	1.6 (0.4–5.6)	1.0 (0.2–5.2)	1.000
**Metabolic**	0.3 (0.1–0.9)	0.0 (0.0–1.1)	0.0 (0.0–1.1)	0.0 (0.0–1.4)	0.8 (0.1–4.1)	0.0 (0.0–2.8)	0.0 (0.0–2.9)	0.0 (0.0–3.6)	1.000
**Effusion**	0.0 (0.0–0.5)	0.0 (0.0–1.1)	0.0 (0.0–1.1)	0.0 (0.0–1.4)	0.0 (0.0–2.8)	0.0 (0.0–2.8)	0.0 (0.0–2.9)	0.0 (0.0–3.6)	1.000

P–values (Holm–adjusted) represent comparison between breeds.

## Discussion

This study reported the most prevalent disorders recorded in dogs attending primary-care veterinary practices in England as otitis externa, periodontal disease and anal sac impaction, while the most prevalent disorder groups were enteropathic, dermatological and musculoskeletal. The head-and-neck was the most prevalent body location affected, the integument was the most prevalent organ system affected, and inflammation was the most prevalent pathophysiologic process. Some evidence was shown to support higher disorder prevalence in purebred dogs compared with crossbred dogs and for important differences in disorder prevalence between breeds.

The current study was designed to fill a critical data gap relating to disorder prevalence information that has been identified as a constraint to improving dog welfare by effective reform of purebred dog-breeding [20], [21], [22]. Unacceptably high occurrence of inherited disorders in purebred dogs has been discussed since over half a century ago [41], [42], [43], [44], leading to implementation of disease control measures such as defined health schemes [45], [46], [47], [48] and revised KC recommendations and rules for registration and showing [44], [49]. However, the current state and predicted trajectory of purebred dog health remain contentious despite these and other ongoing health measures, suggesting that these earlier breeding reforms that were developed without access to prioritisation information on the overall disorder burden may at best have been sub-optimal, and potentially even counter-productive [50].

Primary-care veterinary clinical data have been proposed as a superior data resource for clinical research in dogs [12], [20]. Although useful, alternative data sources including referral practice data [51], [52], [53], pet insurance databases [27], official health schemes [54], [55], [56] and large scale questionnaire surveys [26], [57], [58], [59] are reported to suffer many limitations for the generation of prevalence values that can be generalised to the wider dog population. Analyses based on primary-care veterinary EPR data benefit from open-ended data collection allowing generation of stronger evidence from cohort compared with cross-sectional study designs [60], [61], [62]. Selection bias is reduced by merging data collected from a miscellany of practices [63] and recall and misclassification biases are reduced by collection of clinical notes recorded contemporaneously by veterinary clinicians during episodes of care [64]. Veterinary primary-care denominator populations are well-characterised demographically within PMSs and include all practice-attending animals, whether presenting healthy or sick, linked with comprehensive clinical documentation that facilitates internal validation [27]. Registration databases from primary-care practices are more representative of the national dog population than other databases available for research purposes; 77% of UK dogs are registered with a veterinary practice compared with just 42% of UK dogs that are insured and 31% of UK dogs that are registered with the KC [2].

Previous large-scale studies using primary-care practice clinical data have been variably successful and have encountered problems with sustainability. A cross-sectional study of paper-based clinical records for 7,146 dogs from eight UK practices described demographic and morbidity results but concluded that direct electronic extraction of clinical data and implementation of standardised coding for breeds and disorders were required to sustain long-term data collection [65]. In the US, the National Companion Animal Study (NCAS) reported overall disorder prevalence values using electronic records from 86,772 dogs attending 63 private practices. However, prevalence estimation was based only on the 36% of animals that had at least one coded disorder term recorded and the full clinical notes were not accessible for case-finding and internal validation exercises [66]. The National Companion Animal Surveillance System (NCASP) was established using EPR data from over 500 Banfield Pet Hospitals, but this system focused on the threat of emerging infection, terrorist attack or natural disaster rather than disorder prevalence [67] and has since been discontinued [68].

A standardised veterinary lexicon is critical for large-scale epidemiological application of secondary clinical data [52], [65], [69], [70]. The VeNom codes [30] offers an open-access veterinary nomenclature that has been developed collaboratively between university and primary-care practice groups and facilitates both direct coding by attending clinicians at the point of clinical care and also retrospective coding by researchers during analysis. The VeNom coding ontology that is made available for point-of-care coding defines multiple clinical fields including species (45 terms), dog breeds (767), cat breeds (101), presenting complaints (201), diagnostic tests (39), diagnoses (2,291) and procedures (780).

The current study indicated that otitis externa (10.2%), periodontal disease (9.3%), anal sac impaction (7.1%) and overgrown nails (7.1%) were the most prevalent disorders recorded in dogs attending veterinary practices in England. A US primary-care study similarly identified dental calculus (20.5%), gingivitis (19.5) and otitis externa (13.0%) as the most prevalent diagnoses in dogs, but reported the prevalence of anal sac disease at only 2.5%, and did not even include nail disorders within the common disorders diagnosed [70]. An under-developed coding system, inconsistent case definitions and selection bias from inclusion of only the one-third of animals that had at least one coded diagnosis term within the US study may explain these differing prevalence trends and underscores the importance of standardised coding systems for reliable comparisons between studies. The high frequency of dental disease reported in the US study may have resulted from inclusion of animals with any recorded dental abnormality, regardless of severity. By contract, the current study aimed to report the occurrence of dental disorders that currently warranted treatment in the opinion of the attending clinician. Study-inclusion of dental abnormalities of any nature provides information on the summative effects from both current and potential future clinically-significant dental disease whereas including just current clinically-significant cases provides evidence on the current welfare implications of dental disease. Both approaches have merit and add to our understanding of the substantial clinical relevance of dental disorders to the health and welfare of dogs. A UK primary-care study using paper-based clinical records identified the most prevalent disorders of dogs as overgrown nails (2.7%), ascarid worm problems (2.3%), anal sac impaction (2.1%), dental calculus (1.8%), fleas (1.8%), bacterial otitis externa (1.7%), waxy otitis externa (1.2%), diarrhoea/vomiting (1.0%) and *Otodectes* otitis externa (0.9%) [65]. Although the predominance of aural, nail, anal sac and dental disorders identified was consistent with the current study, the older study reported *prevalence per consultation* values, leading to apparently lower prevalence values than the current study that reported *period prevalence per dog*. The substantially lower prevalence of parasitic disorders reported in the current study may also reflect increasing adoption and effectiveness of prophylactic parasiticides in the intervening fifteen years since the previous study [71], [72].

Although diagnosis-level disorder terms are useful to describe disorders at their precision of clinical diagnosis, sole reliance on these terms for research may mask important underlying disorder concepts because of fragmentation into multiple terms along diagnostic pathways. The current study grouped clinically-related diagnosis-level terms (430 unique terms) into appropriate, composite mid-level disorder terms (54 unique terms) for further analysis. Selection of cut-off points for amalgamation along diagnostic precision pathways aimed to optimise interpretability whilst still retaining adequate precision [73]. The predominant mid-level disorders (enteropathic, dermatological, musculoskeletal and aural) differed from the predominant diagnosis-level disorders (otitis externa, periodontal disease, anal sac impaction, overgrown nails), suggesting that such hierarchical analysis can offer useful insights that may otherwise be missed.

Syndromic surveillance is based on clinical features that are discernible even from early presentation and are not dependent on complete or even correct diagnosis for elucidation of diagnostic patterns [74]. Although veterinary clinical diagnostic accuracy may have improved over recent years, diagnostic discrepancies have been identified in 15% of cases undergoing necropsy [75]. Syndromic surveillance has been applied within human bioterrorism surveillance [76] and for analysis of canine insurance data [77], [78]. The three syndromic classification systems used in the current study (body location, organ system and pathophysiology) were selected for their potential welfare importance via breed conformation and genetic effects [15]. The syndromic coding system used in the current study was adapted from VeNom codes and other published veterinary lexicons in line with the disorder types recorded within the study [25], [79]. Progression towards a standardised syndromic terminology would facilitate future inter-study comparisons and meta-analyses [80].

The results from syndromic analyses in the current study identified the most prevalent body locations affected by disorders in dogs as the head-and-neck (32.8%), abdomen (25.6%) and limb (17.5%). Morphologic diversity between breeds resulting from artificial selection towards the extremes of breed standard morphometrics [81] has been associated with conformational predisposition for disorders [15], [20]. The predominance of disorders identified affecting the head-and-neck reaffirm the importance of this body area to dog health [82].

The most affected organ systems identified by the current study were the integument (36.3%), digestive (29.5%) and musculoskeletal (14.8%). Swedish insurance data analysis similarly identified the most prevalently affected organs systems as the integument (3.2%), gastrointestinal (2.7%) and genital (2.5%) [83]. A consistently high prevalence reported by these studies for disorders affecting the integument and digestive systems suggests the importance of clinical emphasis on maintaining the health of these systems.

The current study identified inflammation (32.1%), mass/swelling (16.1%) and trauma (14.3%) as the most prevalent pathophysiologic processes affecting dogs. Similarly, a Swedish insurance study identified inflammation (5.4%), symptomatic (3.0%), trauma (2.7%) and neoplasia (2.1%) as the pathological processes with the highest risk of morbidity [83]. Although an essential adaptive response to injury, inflammation can behave both physiologically (restoring homeostasis) and pathologically (contributing to ongoing disease) [84]. The preponderance of inflammatory disorders affecting dogs identified by the current study suggests welfare gains from increased awareness by owners of judicious use of anti-inflammatory medications and also the value from ongoing research to better harness the healing aspects of inflammation while limiting detrimental effects [85].

The current study hypothesised that purebred dogs have higher prevalence of common disorders compared with crossbreds. This hypothesis was founded on reports and studies that concluded substantial detriment to purebred dog welfare from increasing inherited health problems induced by inbreeding and selection for extreme morphologies [15], [16], [20], [21], [22]. The study hypothesis was tested by comparing prevalence values between purebreds and crossbreds for each of the twenty most prevalent diagnosis-level and mid-level disorders and for all syndromic presentations. Purebreds showed significantly higher prevalence values for 13 of the 84 (15.5%) disorders and syndromes evaluated. No instances were identified in which prevalence values were significantly higher in crossbred than in purebred dogs. These results provided moderate evidence for higher disorder prevalence in purebreds compared with crossbreds. However, additional analyses of severity and duration data for these disorders would enable a more comprehensive understanding of health disparities between the groups [23].

Failure to show overwhelming evidence for disorder disparity between purebred and crossbred dogs appears initially at odds with the large body of literature apparently to the contrary [20], [21], [22], [86], [87]. There are a number of possibilities for this dissonance. Breed-specific conformational disorders within purebreds may be under-reported or under-recognised by both veterinarians and owners because ‘normal for breed’ may have become confused with ‘normal’ [88]. A study of dogs clinically diagnosed with brachycephalic obstructive airway syndrome (BOAS) identified that 58% of owners reported these dogs not to have ‘breathing problems’ [82]. Purebred and crossbred dog categories comprise heterogeneous mosaics of size, shape and genetics. Merging this variation into single categories may have masked important effects related to specific conformational, physiological or behavioural features. Analyses of purebred or crossbred subgroups based on breed, behaviour or body attributes may better elucidate important health hazards, benefits and associations.

Purebred dogs comprise 75-80% of the overall UK dog population [3], [28], suggesting that a high proportion of crossbreds are likely to be first or second filial offspring from purebred progenitors and could be reasonably expected to show conformational and polygenic disorder occurrence at the midpoint between the values for their parent breeds, with any additional health benefits in crossbreds resulting from hybrid vigour effects [89]. From this perspective, the less-than-overwhelming evidence provided by the current study for substantially lower prevalence values in crossbred compared with purebred dogs does not refute claims in the literature of rising prevalence values for inherited disorders within purebred dogs. Instead, this suggests that the overall disorder burden within crossbred dogs may reflect the overall disorder burden in purebreds at any point in time. For optimal understanding, disorder prevalence in purebreds should be quantified by analysing cohort health data to identify trends over time.

The most prevalent disorders identified in dogs within the current study were complex disorders that have multiple interacting environmental and genetic casual factors [90]: otitis externa [91], periodontal disease [92], anal sac disorders [93], nail disorders [94], [95], degenerative joint disease [96], diarrhoea [97], [98], obesity [99], traumatic injury [100], conjunctivitis [101], vomiting [101], [102] and heart murmur [103], [104]. It may be useful for canine health research to move away from viewing individual disorders as necessarily either inherited or non-inherited [105] and towards an acknowledgement of relevant roles for both genetic and environmental components in the majority of canine disorders [106], [107], [108]. This acceptance will improve decision-making on effective disease-control and breeding programs [109]. Application of estimated breeding values (EBVs) developed from summative health information derived from a range of sources, including health schemes and veterinary primary-care data, could contribute integrally to novel disorder-control programs [14], [110], [111].

A large body of literature supports the existence of disorder predispositions affecting most dog breeds [15], [16], [112]. Despite inclusion of just seven breeds in the current analysis, breed associations were identified for 33.3% (28/84) of the disorders and syndromes evaluated (diagnosis-level disorders 20% (5/20), mid-level disorders 40% (8/20) and syndromic terms 34% (15/44)). The high-risk breeds differed considerably between the disorders in the current study, suggesting that rational health control measures should focus on highly-predisposed disorders within at-risk breeds. Future breed-specific studies are recommended to report more precise prevalence estimates and for a wider range of breeds. Early studies could focus on the fourteen high-profile breeds identified by the KC as having higher health risks, mainly due to conformational problems [113].

There were some limitations to the current study. The practices participating in the study formed a single veterinary group that extended across central and south-east England and may not be representative of the overall veterinary practice structure in England. Case definitions and diagnosis recording relied heavily on the clinical acumen and note-making of attending practitioners. The researchers made no attempts to second-guess underlying disorders in cases with presenting signs (e.g. vomiting) recorded *in lieu* of formal diagnoses. Inclusion of umbrella terms such as *road traffic accident* without additional inclusion of the individual specific injuries sustained within the primary event may have reduced the apparent prevalence of fractures and lacerations but avoided multiple counting of disorder events along axes of diagnostic precision. The analyses based on popular breeds were exploratory in nature and should be validated within larger confirmatory studies [114], [115]. Holm adjustments to P-values were used to constrain the number of false-positive findings resulting from interpretation of multiple comparisons [38], [115], [116]. The current study reported prevalence values but effective welfare prioritisation would additionally benefit from the generation of accurate data on disorder severity and duration [117].

## Conclusion

This study describes the most frequently recorded disorders in dogs in England and provides a prevalence baseline against which to measure progress in canine health. The most prevalent disorders recorded in dogs attending primary-care veterinary practices in England were otitis externa, periodontal disease and anal sac impaction, and the most prevalent disorder groups were enteropathic, dermatological and musculoskeletal. The head-and-neck was the body location most frequently affected by the disorders recorded, the integument was the most prevalent organ system affected and inflammation was the most prevalent pathophysiologic process. The study identified some evidence that purebred dogs had higher disorder prevalence compared with crossbred dogs. Substantial variation was shown across breeds in their prevalence of common disorders. These results suggest that breeding reforms should target commonly diagnosed complex disorders that are amenable to genetic improvement on a breed-by-breed basis for the greatest population impact. The prevalence information provided by this study fills a crucial data gap. Future studies of disorder severity and duration would augment the current results and contribute to increasingly effective strategies to improve dog welfare based on disorder prioritisation.
